# Late-onset sepsis and encephalopathy after bicycle-spoke injury: a case report

**DOI:** 10.1186/s12879-019-4082-4

**Published:** 2019-05-28

**Authors:** Ryuichi Takemoto, Yoshitomo Motomura, Noriyuki Kaku, Yuko Ichimiya, Mamoru Muraoka, Shunsuke Kanno, Tamami Tanaka, Yasunari Sakai, Yoshihiko Maehara, Shouichi Ohga

**Affiliations:** 1 Department of Pediatrics, Graduate School of Medical Sciences, Kyushu University, 3-1-1, Maidashi, Higashi-ku, Fukuoka, 812-8582 Japan; 2 Emergency and Critical Care Center, Kyushu University, Fukuoka, Japan

**Keywords:** Sepsis-associated encephalopathy, Ankle injury, Cellulitis, Pathogens, And *Staphylococcus aureus*

## Abstract

**Background:**

Bicycle-spoke injuries rarely cause late complications of infection, including sepsis and sepsis-associated encephalopathy, with appropriate treatments.

**Case presentation:**

We experienced a 2-year-old girl who developed the signs of encephalopathy with fever 6 months after a spoke-injury. On admission, the injured skin was inflamed with cellulitis. The blood culture was positive for methicillin-sensitive *Staphylococcus aureus*. Electroencephalogram showed diffuse slow-wave activity. Diffusion-weighted magnetic resonance imaging detected a high-intensity lesion with decreased diffusivity at the right frontal cortex. She received immunoglobulin and combined antibiotics treatments in the intensive care unit, and successfully overcame the sepsis-associated encephalopathy without neurological impairments.

**Conclusion:**

This is the first report demonstrating that sepsis and its associated encephalopathy occurs in a remote period after the bicycle-spoke injury.

## Background

Bicycle-spoke injuries (BSI) are caused when the passenger’s foot is caught by the spokes in the rotating wheel of bicycle [1]. The outcome of spoke injuries is generally well with the appropriate treatment, and serious complication rarely occurs in the remote period [2]. However, the internal degloving injury on the skin and soft tissue predisposes patients with BSI to the development of sepsis and other systemic infections.

Sepsis-associated encephalopathy (SAE) is a diffuse cerebral dysfunction that occurs secondary to sepsis in the absence of direct central nervous system (CNS) infection. The diagnosis depends on the exclusion of primary CNS infection and other causes of encephalopathy, because of no specific markers available for SAE. The morbidity and mortality increase with the severity of SAE. Thus, early identification and prompt treatment of underlying infection are important [3, 4].

We herein report a case with cellulitis and SAE that developed 6 months after an accident with bicycle spoke-injury.

## Case presentation

**Fig. 1:**
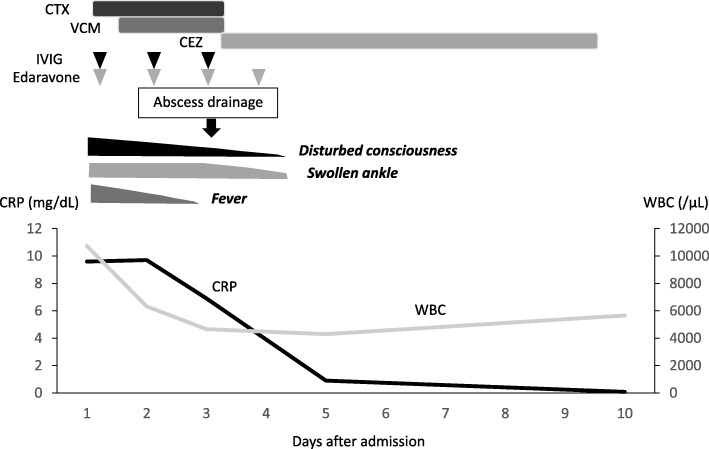
Clinical course after admission. Antibiotics treatments with cefotaxim (CTX), vancomycin (VCM) and cefazolin (CEZ) are shown at the top. Arrowheads indicate intravenously administered immunoglobulin (IVIG) and edaravone. Ameliorating signs of clinical symptoms are shown in the middle. Laboratory data of white blood cell counts (WBC /μl) and C-reactive protein (CRP mg/dl) are line-plotted at the bottom

A 2-year-old girl had an accident of spoke injury. On the day of the accident, she visited a clinic, where she was diagnosed with a laceration on her left ankle. Because the bone fracture was less likely, ultrasonography or x-ray was not examined. She received wound cleaning and an oral antibiotic. However, she stopped visiting the clinic on her parents’ decision after a few days. Six months after the accident, she had a fever at 39 °C, general fatigue and localized pain at the left ankle. She revisited the clinic and received oral third-generation cephalosporin. On the same day (Day 1), she presented with generalized tonic-clonic convulsions for 5 min. The convulsion stopped spontaneously. She was transferred to our hospital because her consciousness remained disturbed after the convulsion. On admission, her body temperature was 39.8 °C, heart rate 160/min, blood pressure 120/82 mmHg, and respiratory rate 50/min. Capillary refilling time was 3 s. Consciousness was evaluated as GCS 7 (E1V2M4). The left ankle was swollen. Laboratory tests showed leukocyte counts of 10,700 /μL with 90% neutrophils. C-reactive protein and procalcitonin were 9.6 mg/dL and 55.5 ng/mL, respectively. Ammonia levels and coagulation studies were normal. The cerebrospinal fluid contained nucleated cells at 1/μL, total protein 17 mg/dL, and glucose 81 mg/dl. IL-6 and IL-8 levels were increased to 37.1 and 455.2 ng/ml in the cerebrospinal fluids, respectively. After the diagnosis of sepsis, she received the administration of cefotaxim and vancomycin. Repeated tests of blood culture proved bacteremia with methicillin- susceptible *Staphylococcus aureus* (MSSA). No pathogens were detected in the cerebrospinal fluid (Fig. 1). The cellulitis on her ankle was thought to be the focus of systemic infection. However, the diagnosis was not confirmed until MRI was taken on day 3 of admission.

**Fig. 2:**
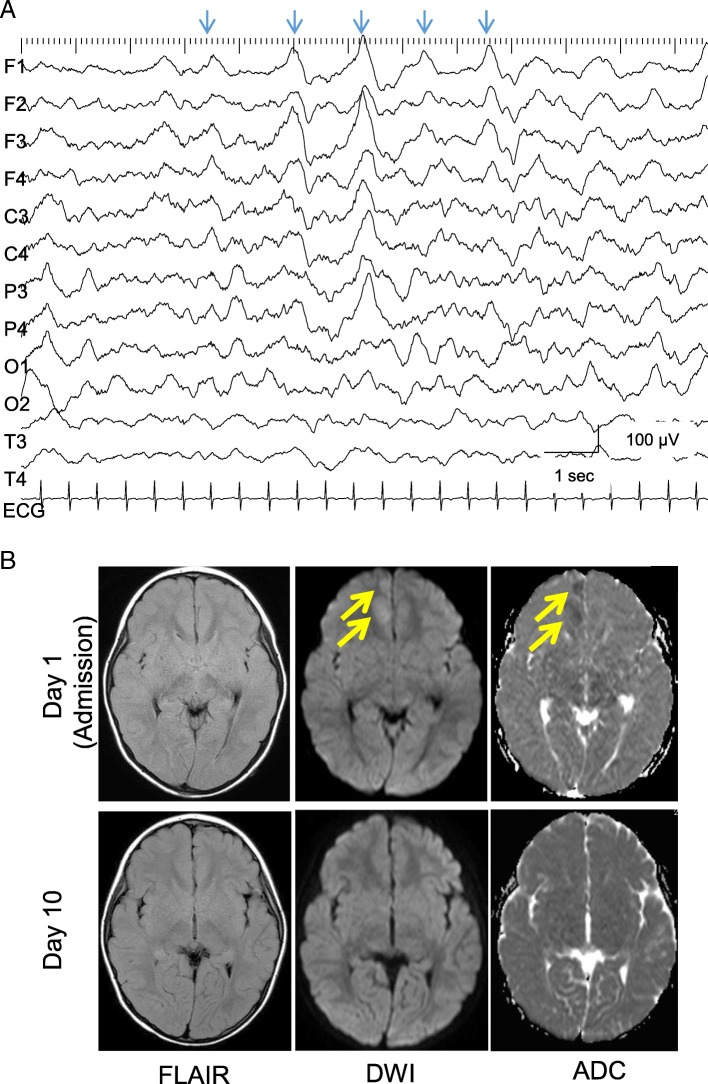
Electrophysiological and neuroimaging findings. **a** An inter-ictal recording of electroencephalogram on day 1. Arrows indicate that irregular, diffuse high-voltage slow waves appear intermittently in the left frontal-dominant manner. **b** Upper: Fluid attenuated inversion recovery (FLAIR), diffusion weighted images (DWI) and apparent diffusion coefficient mapping (ADC) on day 1. Lower: those on day 10. Arrows denote the lesions in the right mesial cortex

Her consciousness did not recover during the next 24 h. Electroencephalograms showed poorly organized background activity, consisting of frontal-dominant, diffuse high-voltage slow waves. Epileptiform discharges were not evident (Fig. 2a). Brain magnetic resonance imaging revealed the T2-prolonged lesions in the mesial frontal cortex of the right hemisphere, accompanying the feature of reduced diffusion on diffusion-weighted imaging (DWI, b factor of 1000 s/mm^2^) and apparent diffusion coefficient mapping (Fig. 2b, upper). Based on the diagnosis of SAE, intensive care was started with 1 g/kg intravenous immunoglobulin (IVIG) for 2 days and 0.5 mg/kg/day edaravone infusion for 4 days. We used both agents because we had not removed the possible complications of SAE with immunocompromised state, autoimmune, hyper-inflammatory or secondary ischemia, including Moyamoya disease [5–7].

**Fig. 3:**
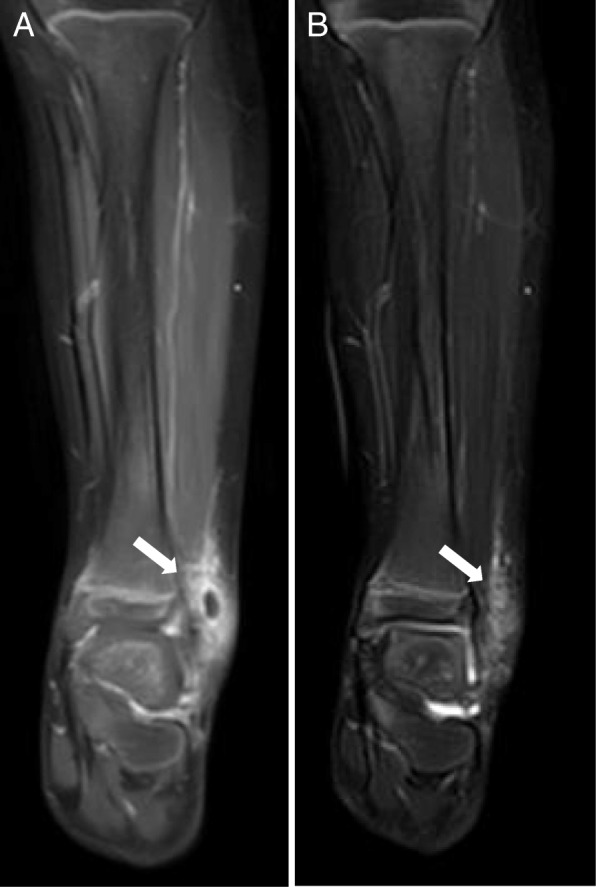
Magnetic resonance imaging of the left lower leg. T1 with enhancement (**a**) and T2-weighted images (**b**) on day 3 are shown. Note that hyper-intense signals (arrows) are present at the left lateral condyle

From the third day of admission, her consciousness began to recover. However, swelling of the left ankle further progressed. T1-weighted image of the left lower leg detected high-intensity signals with enhancement in the adjacent regions of soft tissues Osteomyelitis was not detected (Fig. 3). Subcutaneous abscess was surgically drained, from the culture of which MSSA was also isolated. On the fourth day, the swelling on her ankle improved and her consciousness became clear. On the 10th day after admission, brain MRI showed no abnormal findings (Fig. 2b, lower). She was discharged from our hospital on the 14th day of admission (Fig. 1). Immunological tests did not support evidence for primary immunodeficiency or immunocompromised status (data not shown). She has been fully recovered, and presently attends preschool without any neurological disability.

## Discussion and conclusions

We experienced a case who developed a late-onset staphylococcal infection and encephalopathy after BSI. The soft-tissue injury occurs in all cases with BSI, however, the severity of BSI is often underestimated [2]. The lower extremities are known as the most common site of cellulitis. Notably, the recurrence of cellulitis has been reported to occur in 22 to 49% of affected cases. Among them, 14% of the recurrence was observed in 1 year, while 45% in 3 years. The recurrence typically occurs at the same site as the originally inflamed region [8, 9]. This fact indicates that the recurrence of cellulitis and the following bacteremia may develop several months after the previous injury. Of course, we cannot exclude the possibility that a recent infection was associated with the bacteremia in this case. However, this report provides a better caution that surface injuries could lead to the systemic infection even after 6 months.

SAEs have been reported in children at 4 to 12 years of age. Clinical features of SAE are characterized by the onset with fever and variable levels of brain dysfunction. Altered consciousness could vary from confusion to coma, according to their general conditions [10]. Electrophysiological studies have shown that periodic epileptiform discharges were commonly observed in patients with SAE [11]. Although periodic activity was not clearly demonstrated in our case, synchronized delta activity was observed during the acute phase. Because her consciousness was recovered within 1 week, this intermediate finding might suggest that her brain was less severely damaged by septic condition than those presenting with a complete form of periodic discharges [12].

Neuroimaging features of SAE include ischemic lesions with hyperintense signals on T2-weighted images [13]. Vasogenic edema is also a common finding in patients with SAE, and it has been also reported to indicate the aberrant function of blood-brain barrier. This mechanism explains well the fact that some patients with SAE presented with posterior reversible encephalopathy syndrome [14]. By contrast, our patient showed the sign of localized cytotoxic edema in the frontal cortex. Because it disappeared without leaving the persistent ischemic lesion, we speculated that hemodynamics of the brain was not severely affected during the acute phase. Neuroimaging features differ among patients according to their ages, severity and the time of evaluation.

The pathophysiology of SAE has not been fully understood. A variety of mechanisms have been proposed, including microscopic brain injury, altered cerebral microcirculation or metabolism, aberrant neurotransmission, and inflammatory mediators [3]. Previous studies suggested that biliary tract or intestinal infections were associated with greater risk of SAE [3, 4]. With regard to pathogens, the most commonly implicated organism is *Staphylococcus aureus*, as described in this and previous reports [15]. In the present case, proinflammatory cytokines of IL-6 and IL-8 were elevated in CSF. We therefore considered neuroinflammatory signals to be involved in the pathogenic process of SAE.

In summary, this report provides the first evidence that the spoke injury-associated infection causes the recurrent cellulitis after months and raises the risk for the development of SAE in childhood. Cause-and-effect relationship among the pathogens, cytokines in the cerebrospinal fluid, acute-phase brain dysfunctions and long-term outcomes will be worth investigated for SAE patients in future studies.

## Abbreviations

BSI: Bicycle spoke injury
DWI: Diffusion weighted image
GCS: Glasgow coma scale
IL: Interleukin
IVIG: Intravenous immunoglobulin
MSSA: Methicillin-susceptive *Staphylococcus aureus*
SAE: Sepsis associated encephalopathy
